# Association of Triglyceride‐Glucose Index and Its Related Parameters With Handgrip Strength Asymmetry

**DOI:** 10.1002/jcsm.70165

**Published:** 2026-02-01

**Authors:** Yuan Zhang, Yali Jing

**Affiliations:** ^1^ Department of Endocrinology, Endocrine and Metabolic Disease Medical Center Nanjing Drum Tower Hospital, Affiliated Hospital of Medical School, Nanjing University Nanjing China; ^2^ Branch of National Clinical Research Centre for Metabolic Diseases Nanjing China

**Keywords:** aging population, handgrip strength (HGS) asymmetry, insulin resistance (IR), triglyceride‐glucose (TyG) index

## Abstract

**Background:**

The purpose of this study was to explore the associations of the triglyceride‐glucose (TyG) index and its clinically relevant derivatives with handgrip strength asymmetry (HGS‐A) in a nationally representative cohort of older adults in China.

**Methods:**

Here, we used the data of participants aged between 45 and 80 years who were from wave 2015 of the China Health and Retirement Longitudinal Study (CHARLS). HGS asymmetry was defined as the ratio of nondominant to dominant HGS, categorized into the following four groups: 0.9–1.1 (nonasymmetry), 0.80 ≤ ratio < 0.90 or 1.10 < ratio ≤ 1.20 (mild asymmetry), 0.70 ≤ ratio < 0.80 or 1.20 < ratio ≤ 1.30 (moderate asymmetry) and ratio < 0.70 or ratio > 1.30 (severe asymmetry). Multivariable logistic regression was adopted to assess the association of TyG, TyG‐body mass index (TyG‐BMI) and TyG‐waist‐to‐height ratio (TyG‐WHtR) with HGS asymmetry.

**Results:**

A total of 3521 middle‐aged and elderly individuals (mean age: 59.74 years; 47.9% male) were included, with the mean TyG of 8.733 ± 0.660, mean TyG‐BMI of 243.559 ± 31.120, mean TyG‐WHtR of 4.702 ± 0.733. The mean HGS ratio was 0.97 ± 0.16, and participants were classified into the following four groups: nonasymmetry (*n* = 2174), mild asymmetry (*n* = 972), moderate asymmetry (*n* = 237) and severe asymmetry (*n* = 138). Overall, the prevalence of HGS asymmetry was 38.26%. The prevalence of severe asymmetry increased across TyG quartiles from 3.7% to 4.5% (Q1–Q4, *p* = 0.018). TyG‐WHtR showed increasing prevalence of severe asymmetry from 4.9% to 5.1% across quartiles (*p* = 0.042). For TyG‐BMI, it exhibited an inverse relationship with severe asymmetry prevalence decreasing from 5.3% to 2.4%. Multivariable‐adjusted models confirmed that TyG‐WHtR demonstrated the strongest effect size with Q2 and Q3 associated with significantly increased odds of asymmetry (OR = 1.507 and 1.437, respectively). TyG‐BMI showed a protective effect with higher quartiles associated with reduced odds (Q2–Q4 OR range: 0.973–0.984).

**Conclusions:**

Higher levels of TyG‐WHtR and lower levels of TyG‐BMI are both associated with a higher prevalence and severity of HGS asymmetry in middle‐aged and older Chinese adults.

## 1 Introduction

Muscle strength in adults tends to decrease with advancing age [1]. Handgrip strength (HGS) has been widely recognized as a robust biomarker of overall muscular strength and a powerful predictor of adverse health outcomes including weakness, frailty, disability and all‐cause mortality [2, 3]. Increasing evidence indicated that individuals with frailty or weakness, as a multisystemic age‐related syndrome, were prone to falls, disability, hospitalization and premature death, contributing to a substantial social and economic burden [4, 5]. Recently, the growing studies suggested that muscle strength asymmetry (HGS‐A) may signal deteriorating muscle health prior to a decline in maximal muscle strength [6]. Handgrip strength asymmetry, the disparity in handgrip strength between the dominant and nondominant hands, could reflect another dimension of strength capacity, namely, strength imbalance, which is closely connected with disparities or potential neuromuscular and musculoskeletal disorders [7]. Previous researches have reported that HGS asymmetry was associated with the higher risk of hip fracture, cognitive impairment, functional disability [7, 8], as well as may accelerate time to mortality in the elderly [9]. Despite its importance, the underlying metabolic factors contributing to HGS‐A remain poorly understood.

The triglyceride‐glucose (TyG) index, a robust surrogate marker for insulin resistance (IR) that integrates triglyceride (TG) and fasting plasma glucose (FPG) levels, has been increasingly recognized for its role in predicting metabolic dysfunction and related chronic diseases, including diabetes, cardiovascular disease and nonalcoholic fatty liver disease [10]. Beyond systemic metabolic effects, emerging evidence highlighted the essential role of IR in musculoskeletal health. The dual impact of IR on muscle protein homeostasis can lead to progressive muscle wasting, reduced muscle strength and sarcopenia, which may contribute to inter‐extremity muscular asymmetry by differentially affecting muscle integrity or neural control in the dominant versus nondominant extremities [11, 12, 13]. For example, IR‐induced intramuscular lipid accumulation may exhibit uneven distribution in the functionally active dominant extremity or nondominant extremity subjected to greater static loads, thereby predisposing to asymmetry [14, 15]. Furthermore, individuals with IR often exhibit impaired insulin‐stimulated vasodilation in skeletal muscle, which may exacerbate bilateral functional asymmetry by compromising extremity‐specific blood perfusion and local nutrient delivery [16, 17]. Additionally, considering that IR often coexists with obesity in the population, and the two exacerbate metabolic disorders through a synergistic effect, we also simultaneously examine the effects of important derivative indices of the TyG index: triglyceride‐glucose‐body mass index (TyG‐BMI) and triglyceride‐glucose‐waist‐to‐height ratio (TyG‐WHtR). TyG‐BMI integrates BMI to reflect the combined effects of IR and systemic obesity, while TyG‐WHtR combines abdominal obesity to assess metabolic status. Obesity is a driving factor in chronic low‐grade inflammation and muscle factor imbalance, accelerating muscle dysfunction and thereby driving the occurrence of muscle strength asymmetry. Additionally, the magnitude of adiposity is associated with postural changes and unilateral muscle strain, which may exacerbate grip asymmetry through biomechanical stress or neuromuscular coordination defects [18, 19].

Previous studies have predominantly focused on the associations between the TyG index and its related parameters with muscle mass or global handgrip strength, confirming the impact of metabolic dysregulation on total muscle volume and overall strength [20, 21]. However, HGS‐A, as a sensitive indicator of asymmetric muscle function, can more early reflect subtle abnormalities such as imbalanced muscle neural control and localized strength decline, yet it has received insufficient attention in existing research. China's burgeoning older adult population accompanies a critical public health challenge, with the prevalence of skeletal or muscle diseases and metabolic disorders rising in parallel. Therefore, this study aims to investigate the association of TyG index, TyG‐BMI and TyG‐WHtR with HGS‐A in middle‐aged and older Chinese adults, providing novel evidence linking metabolic dysregulation to muscular asymmetry, potentially identifying new insights for preventing age‐related musculoskeletal decline.

## 2 Methods

### 2.1 Study Population

**FIGURE 1:**
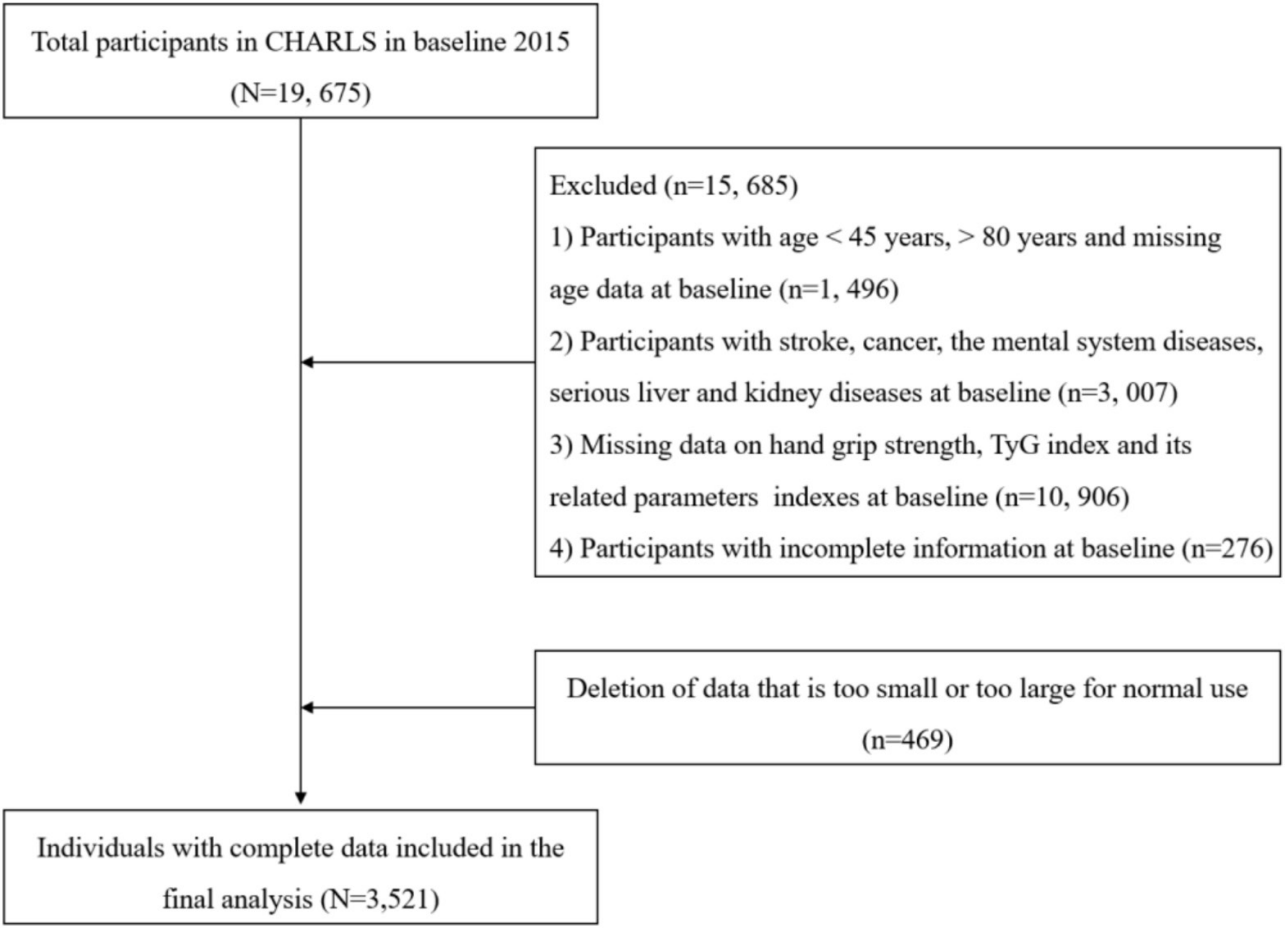
The flow chart of the included participants in this study.

The China Health and Retirement Longitudinal Study (CHARLS) is a large‐scale interdisciplinary survey project hosted by the National Development Institute of Peking University and carried out by the China Social Science Survey Center of Peking University [22]. For this cross‐sectional analysis, data of the population from the 2015 CHARLS cohort were analysed, and the data are available online at https://charls.pku.edu.cn/. Declaration of Helsinki and approved by the Peking University Institutional Review Board (IRB00001052‐11015). All participants signed an informed consent. Participants who officially registered participants in the CHARLS 2015 wave were included if they were aged ≥ 45 years and ≤ 80 years, with complete and reasonable anthropometric measurements (WC, BMI), available handgrip strength evaluations, sufficient biochemical data and complete demographic/health records. Participants meeting any of the following exclusion criteria were omitted: age<45 years or > 80 years, incomplete and extreme anthropometric measurements, missing handgrip strength evaluations, insufficient biochemical data or incomplete demographic/health records. The detailed flowchart of the participants' selection process was displayed in Figure 1.

### 2.2 Assessment of HGS and HGS Asymmetry

Before the measurement of handgrip strength, participants were asked, ‘Did your hand have pain, injury, surgery, swell or inflammation?’ If the answers were ‘no’ and the participants were willing to conduct the handgrip strength measurement, then they were instructed to test the handgrip strength. HGS was measured using a Yuejian WL‐1000 mechanical dynamometer (Nantong, China) [22]. Handgrip strength (unit: kg) was measured in the dominant hand and nondominant hand, with the participant squeezing the dynamometer as hard as possible. Each participant was tested in duplicate for both hands by holding the dynamometer at a right angle (90°) [2]. If the subject was unable to complete the test with either hand (i.e., one hand had a maximum HGS of zero), the HGS was labelled ‘missing.’ HGS values < 28 kg for male participants and < 18 kg for female participants were considered low HGS [1, 2].

The percent difference in HGS between hands is calculated as the ratio of the maximal nondominant HGS and maximal dominant HGS (nondominant HGS/dominant HGS, non–D‐HGS/D‐HGS) [23]. We used three different cutoff values of HGS ratio to define asymmetry, including 10%, 20% and 30%. Specifically, mild asymmetry was defined as 0.80 ≤ ratio < 0.90 or 1.10 < ratio ≤ 1.20 (10%–20% difference), moderate asymmetry was defined as 0.70 ≤ ratio < 0.80 or 1.20 < ratio ≤ 1.30 (20%–30% difference) and severe asymmetry was defined as ratio < 0.70 or ratio > 1.30 (> 30% difference) [24].

### 2.3 Calculation of TyG, TyG‐BMI and TyG‐WHtR

These indexes were obtained by using the following formulas [25]: (1) TyG = Ln[TG (mg/dL) × FPG (mg/dL)/2]; (2) TyG‐BMI = TyG × [weight (kg)/height^2^ (m^2^)]; (3) TyG‐WHtR = TyG × [WC (cm)/height (cm)].

### 2.4 Covariates

The following potential covariates were considered: (1) demographic and lifestyle data: age, gender, smoking, drinking and hypertension; (2) body measurements: height, weight, waist, BMI and WHtR; (3) laboratory test data: blood urea nitrogen (BUN), serum creatinine (SCr), serum uric acid (SUA), FPG, haemoglobin A1c (HbA1c), total cholesterol (TC), TG, high‐density lipoprotein cholesterol (HDL‐C), low‐density lipoprotein cholesterol (LDL‐C), C‐reactive protein (CRP), haemoglobin (HGB) and cystatin C (Cys‐C).

### 2.5 Statistical Analysis

Stata 16.0 was used to clean the CHARLS 2015 data. All data were examined by adopting the program SPSS Statistics software Version 27.0 (IBM SPSS Inc., Chicago, Illinois, USA). The results of the following analyses are derived from complete case data and descriptive analysis was adopted to present the baseline characteristics of the analytical sample. Baseline characteristics were compared among subjects without and with HGS asymmetry of different degrees. The continuous variables in accordance with normal distribution were expressed by (mean ± standard deviation), and the comparison between groups was expressed by ANOVA, while continuous variables with a nonnormal distribution were expressed by medians (interquartile ranges [IQRs]), and the comparison among 4 groups was expressed by the Kruskal–Wallis test. The categorical variables were reported in the form of percentages and comparative analyses of differences were performed using the chi‐square test. The correlation between the ratio of non–D‐HGS/D‐HGS and TyG, TyG‐BMI and TyG‐WHtR was assessed by Pearson's correlation coefficient. Then, the multivariable logistic regression was constructed to identify the relationship of HGS‐A with TyG, TyG‐BMI and TyG‐WHtR, and three models were fitted. The results were evaluated within a 95% confidence interval (CI) and at a significance level of a two‐sided *p* value less than 0.05. *p* value < 0.05 was considered statistically significant.

## 3 Results

### 3.1 Baseline Characteristics

This study included 3521 eligible individuals (mean age 59.74 years, male 47.9%) with a mean nondominant HGS/dominant HGS level of 0.966 ± 0.161. The participants were divided into four groups based on HGS ratio level, including the 0.9–1.1 group (*n* = 2174), the 0.80 ≤ ratio < 0.90 or 1.10 < ratio ≤ 1.20 group (*n* = 972), the 0.70 ≤ ratio < 0.80 or 1.20 < ratio ≤ 1.30 group (*n* = 237) and the ratio < 0.70 or ratio > 1.30 group (*n* = 138). Table 1 summarizes the characteristics of the participants. A total of 38.26% of individuals had HGS asymmetry, and 28.63% had low HGS. Participants in the HGS ratio level > 1.3 or < 0.7 group had the lowest level of BMI, TyG‐BMI and HGS. Moreover, there was a notably significant difference in TyG, TyG‐BMI and TyG‐WHtR among all groups (*p* < 0.05). Additionally, the proportions of male participants, alcohol consumption and smoking status, as well as the levels of age, BMI, WHtR, HDL‐C, CRP, HGB and Cys‐C were observed to have significant differences among the four groups (*p* < 0.05).

**TABLE 1:** Baseline characteristics of the study participants.

Variable	All (*N* = 3521)	HGS ratio 0.9–1.1 (*n* = 2174)	HGS ratio > 1.1, <0.9 (*n* = 972)	HGS ratio > 1.2, <0.8 (*n* = 237)	HGS ratio > 1.3, <0.7 (*n* = 138)	*p*
Age (year)	59.74 (52.00, 66.00)	59.35 (52.00, 66.00)	60.15 (53.00, 66.00)	60.83 (54.00, 67.00)	60.83 (61.00, 66.00)	0.005
Male (*n*, %)	1687, 47.9%	1170, 53.8%	328, 33.7%	78, 32.9%	111, 62.4%	<0.001
BMI (kg/m^2^)	27.89 ± 2.88	27.97 ± 2.89	28.05 ± 2.84	27.49 ± 2.76	26.43 ± 2.64	<0.001
WHtR	0.537 ± 0.061	0.529 ± 0.062	0.552 ± 0.060	0.548 ± 0.054	0.532 ± 0.054	<0.001
Alcohol consumption (*n*, %)	1465, 41.6%	974, 44.8%	330, 34.0%	80, 33.8%	81, 45.5%	<0.001
Smoking status (*n*, %)	1584, 45.0%	571, 26.3%	290, 29.8%	67, 28.3%	60, 33.7%	<0.001
Hypertension (*n*, %)	988, 28.1%	571, 26.3%	290, 29.8%	67, 28.3%	60, 33.7%	0.056
BUN (mg/dL)	15.66 ± 4.60	15.55 ± 4.32	15.81 ± 4.75	15.52 ± 4.22	16.29 ± 6.93	0.86
SCr (mg/dL)	0.80 ± 0.23	0.80 ± 0.19	0.78 ± 0.27	0.77 ± 0.17	0.85 ± 0.40	<0.001
SUA (mg/dL)	4.77 ± 1.43	4.78 ± 1.43	4.74 ± 1.45	4.66 ± 1.36	4.88 ± 1.33	0.269
FPG (mg/dL)	106.33 (90.09, 109.91)	105.61 (90.09, 108.99)	106.66 (91.08, 110.88)	109.66 (91.26, 109.91)	108.88 (90.09, 111.71)	0.069
HbA1c (%)	5.61 (5.10, 5.90)	5.59 (5.1, 5.9)	5.62 (5.1, 5.9)	5.69 (5.2, 6.0)	5.59 (5.0, 5.8)	0.22
TC (mg/dL)	185.98 ± 38.50	184.77 ± 38.60	188.24 ± 37.59	192.34 ± 42.07	179.95 ± 35.71	0.001
TG (mg/dL)	142.07 (81.42, 171.68)	139.04 (80.53, 168.14)	147.69 (85.84, 183.19)	146.15 (78.32, 178.32)	143.41 (79.43, 176.55)	0.07
HDL‐C (mg/dL)	49.12 ± 12.25	49.15 ± 12.05	48.86 ± 12.72	51.17 ± 12.41	47.43 ± 11.58	0.01
LDL‐C (mg/dL)	107.51 ± 33.29	107.05 ± 33.67	108.67 ± 32.52	109.91 ± 34.00	103.60 ± 31.55	0.088
CRP (mg/l)	2.62 (0.70, 2.40)	2.50 (0.7, 2.2)	2.73 (0.7, 2.6)	3.16 (0.7, 2.5)	2.77 (0.6, 2.37)	0.028
HGB (g/dL)	13.92 (12.80, 15.00)	13.97 (12.8, 15.1)	13.81 (12.7, 14.8)	13.75 (12.5, 14.6)	14.05 (12.8, 15.2)	0.003
Cys‐C (mg/L)	0.90 ± 0.26	0.89 ± 0.24	0.92 ± 0.28	0.91 ± 0.27	0.95 ± 0.30	0.012
TyG	8.733 ± 0.660	8.710 ± 0.650	8.774 ± 0.666	8.763 ± 0.697	8.759 ± 0.684	0.048
TyG‐BMI	243.559 ± 31.120	243.591 ± 30.551	246.318 ± 32.012	240.974 ± 31.536	231.538 ± 29.681	<0.001
TyG‐WHR	4.702 ± 0.733	4.623 ± 0.726	4.860 ± 0.730	4.809 ± 0.682	4.682 ± 0.743	<0.001
Left HGS	30.002 ± 9.989	31.851 ± 10.029	28.284 ± 8.626	25.532 ± 9.371	22.748 ± 10.444	<0.001
Right HGS	31.338 ± 10.142	32.384 ± 10.180	30.798 ± 9.353	29.314 ± 10.176	26.646 ± 10.677	<0.001
HGS ratio	0.966 ± 0.161	0.986 ± 0.053	0.927 ± 0.126	0.900 ± 0.218	1.039 ± 0.557	<0.001
Low HGS (*n*, %)	1008, 28.63%	529, 24.3%	257, 26.4%	91, 38.4%	131, 73.6%	<0.001

**Abbreviations:** BMI, body mass index; BUN, blood urea nitrogen; CRP, C‐reactive protein; Cys‐C, cystatin C; FPG, fasting plasma glucose; HbA1c, haemoglobin A1c; HDL‐C, high‐density lipoprotein cholesterol; HGB, haemoglobin; HGS, handgrip strength; LDL‐C, low‐density lipoprotein cholesterol; SCr, serum creatinine; SUA, serum uric acid; TC, total cholesterol; TG, triglyceride; TyG, triglyceride‐glucose; TyG‐BMI, TyG‐body mass index; TyG‐WHtR, TyG‐waist‐to‐height ratio; WHtR, weight‐height ratio.

### 3.2 Correlations Between the HGS Ratio and TyG, TyG‐BMI and TyG‐WHtR

As shown in Table 2, in all subjects, the HGS ratio is negatively correlated with TyG (*r* = −0.061, *p* < 0.001), TyG‐BMI (*r* = −0.317, *p* < 0.001) and TyG‐WHR (*r* = −0.14, *p* < 0.001). In subgroups categorized by sex, there is a significantly negative correlation between the HGS ratio and TyG‐BMI in males (*r* = −0.269, *p* < 0.001). In females, the notably negative correlation was observed in the HGS ratio with TyG (*r* = −0.057, *p* = 0.013), TyG‐BMI (*r* = −0.13, *p* < 0.001) and TyG‐WHR (*r* = −0.079, *p* = 0.001).

**TABLE 2:** Correlations between the HGS ratio and TyG, TyG‐BMI and TyG‐WHtR.

HGS ratio	All		Male		Female	
	*r*	*p*	*r*	*p*	*r*	*p*
Age	0.04	0.016	0.064	0.009	−0.123	<0.001
BMI	−0.348	<0.001	−0.333	<0.001	0.236	<0.001
WHtR	−0.153	<0.001	−0.011	−0.661	−0.068	<0.001
TyG	−0.061	<0.001	−0.034	0.167	−0.057	0.013
TyG‐BMI	−0.317	<0.001	−0.269	<0.001	−0.13	<0.001
TyG‐WHR	−0.14	<0.001	−0.024	0.316	−0.079	0.001
Left HGS	0.201	<0.001	0.065	0.007	0.306	<0.001
Right HGS	−0.167	<0.001	−0.265	<0.001	0.002	0.929

### 3.3 Association of HGS Asymmetry With TyG, TyG‐BMI and TyG‐WHtR

**FIGURE 2:**
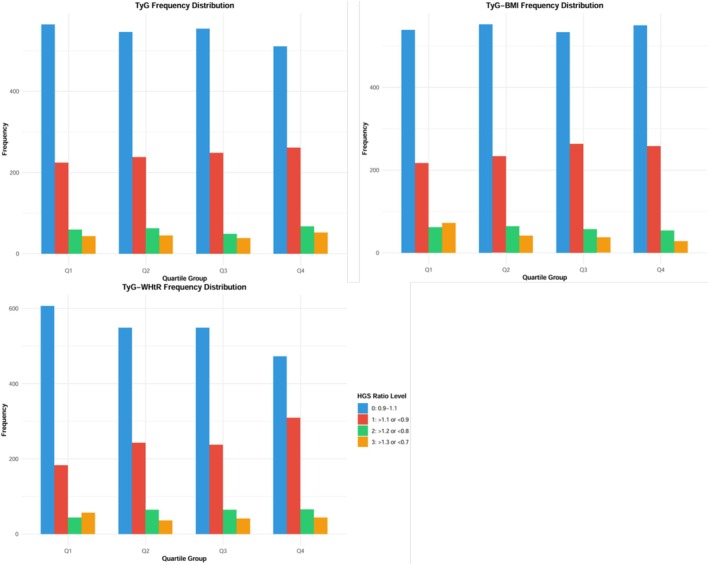
Distribution of TyG, TyG‐BMI and TyG‐WHtR according to HGS ratio level groups.

As presented in Figure 2, when stratified by HGS asymmetry severity using nonoverlapping criteria, clear distribution patterns emerged across TyG index quartiles; distinct patterns emerged for each metabolic index. For the TyG index, a significant positive linear relationship with severe asymmetry was observed. The prevalence of severe asymmetry increased progressively from Q1 to Q4 (3.7%–4.5%, *p* = 0.018). In absolute terms, Q4 contained 52 participants with severe asymmetry compared to 43 in Q1, representing a 21% relative increase. For the TyG‐BMI index, an inverse relationship pattern was identified. The prevalence of severe asymmetry decreased from 5.3% in Q1 to 2.4% in Q4 (*p* = 0.120); in absolute numbers, Q4 contained 28 severely asymmetric individuals compared to 72 in Q1, representing a 61% relative decrease. For the TyG‐WHtR index, the strongest association was demonstrated. The prevalence of severe asymmetry increased significantly from Q1 to Q4 (4.9%–5.1%, *p* = 0.042). Additionally, TyG‐WHtR showed the steepest gradient in distribution, with the Q4 group having 11.6% fewer individuals with non‐HGS asymmetry than Q1 (72.1% vs. 60.5%, *p* < 0.001).

**FIGURE 3:**
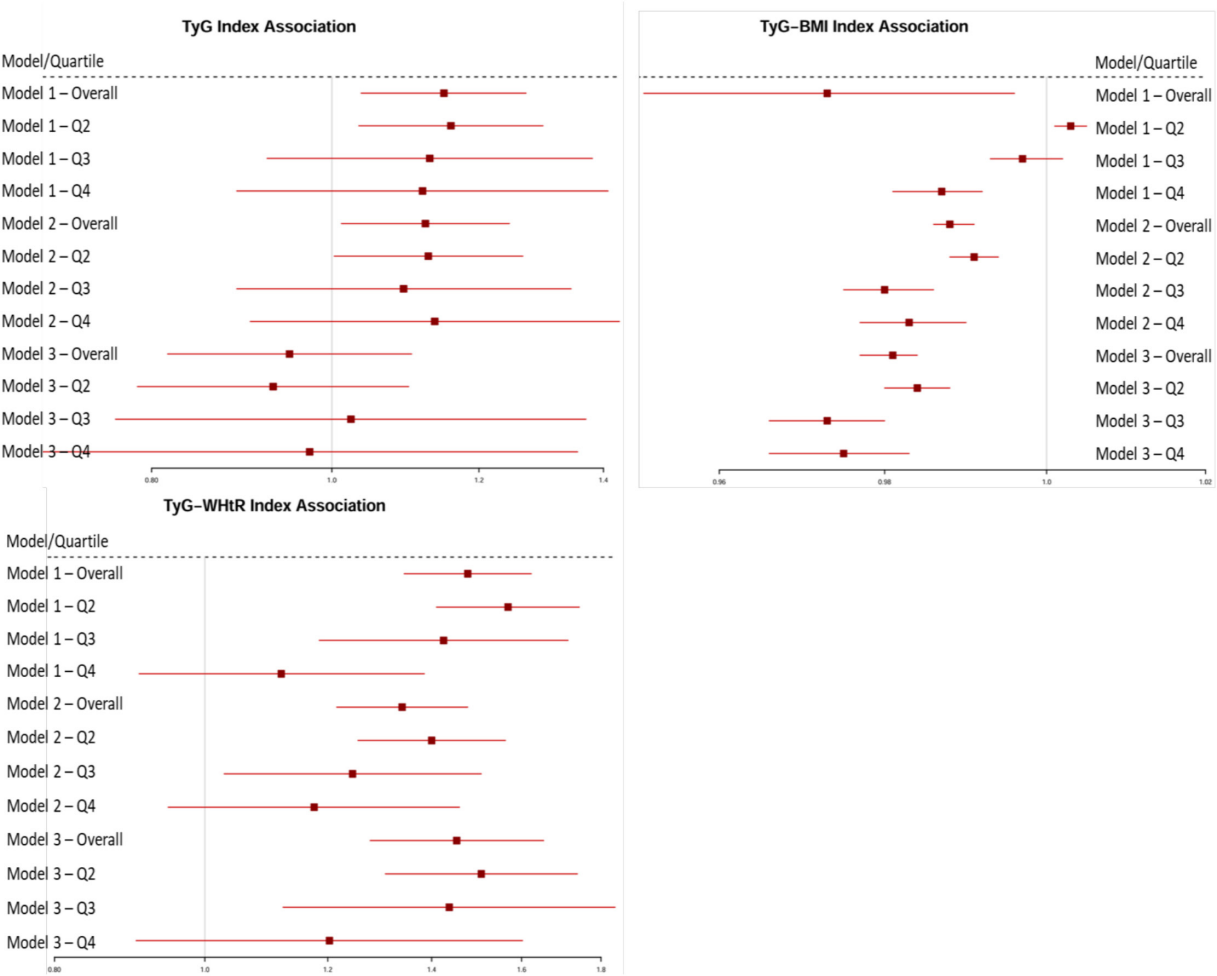
Forest plot of odds ratios for risk of HGS asymmetry with TyG, TyG‐BMI and TyG‐WHtR.

Furthermore, in order to assess the overall HGS asymmetry risk at the TyG, TyG‐BMI and TyG‐WHtR levels and quartiles, logistic analyses were conducted (Figure 3). In the regression analysis of TyG, TyG‐BMI and TyG‐WHtR as the continuous variables, it was shown that TyG‐BMI and TyG‐WHtR levels were significantly associated with the risk of HGS asymmetry both in unadjusted and adjusted models for all subjects in this study, whereas TyG was only associated with the risk of HGS asymmetry in model 1 (Table 3). With the TyG, TyG‐BMI and TyG‐WHtR as the stratified indicators, in the unadjusted model (model 1), for TyG, compared with Q1, Q2 (Q2 vs. Q1, OR, 1.159; 95% CI: 1.034~1.299) kept an independent effect on HGS asymmetry presence; for TyG‐BMI, compared with Q1, Q4 was significantly associated with HGS asymmetry risk, showing an OR of 0.987 (95% CI: 0.981~0.992); and for TyG‐WHtR, Q2 (Q2 vs. Q1, OR, 1.568; 95% CI: 1.410~1.743) and Q3 (Q3 vs. Q1, OR, 1.425; 95% CI: 1.185~1.714) all kept an independent effect on HGS asymmetry presence. After age and gender adjustment (model 2), the analysis revealed that higher TyG‐BMI values were associated with a lower risk of HGS asymmetry, while higher TyG‐WHtR values were associated with an increased risk. After further adjustment in model 2 for other confounding factors, compared to Q1 of TyG‐BMI, subjects in Q2, Q3 and Q4 all showed a potential protective association of HGS asymmetry (OR = 0.984, 0.973 and 0.975, respectively); compared to Q1 of TyG‐WHtR, subjects in Q2 and Q3 had a significantly increased risk of HGS asymmetry (OR = 1.507, 1.437, respectively).

**TABLE 3:** Associations of TyG, TyG‐BMI and TyG‐WHtR levels with the risk of HGS asymmetry.

	Model 1		Model 2		Model 3	
	OR (95% CI)	*p*	OR (95% CI)	*p*	OR (95% CI)	p
TyG	1.149 (1.037,1.272)	0.008	1.123 (1.012,1.246)	0.029	0.949 (0.816,1.104)	0.502
TyG‐BMI	0.973 (0.951,0.996)	0.023	0.988 (0.986,0.991)	<0.001	0.981 (0.977,0.984)	<0.001
TyG‐WHtR	1.477 (1.344, 1.623)	<0.001	1.340 (1.216,1.477)	<0.001	1.453 (1.278,1.653)	<0.001
**TyG**	Model 1		Model 2		Model 3	
	OR (95% CI)	P	OR (95% CI)	P	OR (95% CI)	P
Q1	Reference		Reference		Reference	
Q2	1.159 (1.034,1.299)	0.011	1.127 (1.003,1.267)	0.045	0.93 (0.786,1.1)	0.395
Q3	1.129 (0.923,1.381)	0.236	1.093 (0.889,1.345)	0.398	1.024 (0.765,1.37)	0.875
Q4	1.119 (0.889,1.408)	0.339	1.136 (0.904,1.428)	0.274	0.973 (0.699,1.356)	0.873
**TyG‐BMI**	Model1		Model2		Model3	
	OR (95% CI)	P	OR (95% CI)	P	OR (95% CI)	P
Q1	Reference		Reference		Reference	
Q2	1.003 (1.001,1.005)	0.023	0.991 (0.988,0.994)	<0.001	0.984 (0.980,0.988)	<0.001
Q3	0.997 (0.993,1.002)	0.216	0.980 (0.975,0.986)	<0.001	0.973 (0.966,0.980)	<0.001
Q4	0.987 (0.981,0.992)	<0.001	0.983 (0.977,0.990)	<0.001	0.975 (0.966,0.983)	<0.001
**TyG‐WHtR**	Model 1		Model 2		Model 3	
	OR (95% CI)	P	OR (95% CI)	P	OR (95% CI)	P
Q1	Reference		Reference		Reference	
Q2	1.568 (1.410,1.743)	<0.001	1.400 (1.255,1.562)	<0.001	1.507 (1.307,1.738)	<0.001
Q3	1.425 (1.185,1.714)	<0.001	1.245 (1.029,1.507)	0.024	1.437 (1.123,1.838)	0.004
Q4	1.120 (0.907,1.385)	0.293	1.176 (0.947,1.459)	0.142	1.203 (0.903,1.602)	0.260

*Note:* Model 1 was unadjusted model. Model 2 was adjusted for age (continuous) and gender. Model 3 was further adjusted for alcohol consumption, smoking status, BUN, SCr, TC, HDL‐C, CRP, HGB and Cys‐C.

## 4 Discussion

Leveraging data from representative middle‐aged and older populations in China, we found a higher prevalence of HGS asymmetry (38.26%) than low HGS (28.63%) in the study population, with significant correlations observed between the association of HGS asymmetry and insulin resistance indices, particularly TyG‐BMI and TyG‐WHtR, suggesting that IR may contribute to impaired muscle function symmetry. Multivariable regression analyses further supported TyG‐BMI and TyG‐WHtR as independent predictors of HGS asymmetry, revealing that obesity‐related metabolic disturbances, rather than hyperglycaemia or dyslipidaemia alone, may play a more critical role in muscle function imbalance.

Handgrip strength asymmetry emerges as a more sensitive early biomarker of health decline than handgrip strength alone. Unlike HGS, which primarily reflects muscle strength, HGS asymmetry captures neuromuscular coordination and neural integrity, with childhood‐onset asymmetry persisting into adulthood despite weak tracking, suggesting developmental origins [26]. Longitudinal studies showed that HGS asymmetry independently predicts neurocognitive decline (71% higher hazard in Chinese cohorts) and functional disability (2.67‐fold sarcopenia odds), even when HGS is normal [27]. Thus, HGS asymmetry might offer a modifiable, early intervention target for mitigating disability and cognitive decline, warranting inclusion in geriatric and neurological assessments beyond low HGS. Muscle, as an important endocrine organ, is vital for metabolism and physiology and plays a crucial role in insulin‐mediated glucose disposal [28]. HGS asymmetry is prevalent in older adults and is associated with many adverse outcomes, such as falls, metabolic disease and mortality, demonstrating the value of HGS asymmetry research. Therefore, it is critical to maintain an appropriate level of muscle function for health [29, 30]. IR has been unequivocally established as a pivotal contributor to the pathogenesis of possible weakness or sarcopenia; poor IR may promote muscle protein degradation and impede protein synthesis, leading to loss of muscle mass or strength, which may exacerbate debilitation and associated metabolic disorders, and contribute to a reduction in muscle mass and strength, accelerating the frailty progression and physical function decline, severely affecting patients' disease prognosis and quality of life [31]. Some studies suggested that IR may lead to low muscle function via mechanisms, such as systemic inflammation, oxidative stress, and declining vascular function. Firstly, muscle is the major glucose consumer after a meal, supporting that a block in muscle glucose uptake could lead to increased blood glucose and hyperinsulinemia. The main insulin‐stimulated glucose transporter glucose transporter type 4 (GLUT4) is highly expressed in differentiated muscle tissue, and GLUT4‐mediated glucose transport in muscle is essential to the maintenance of normal glucose homeostasis [32]. H. Shoyhet et al. demonstrated that GLUT4 overexpressing skeletal muscle tissue can improve glucose homeostasis and retain the shape‐memory properties and viability of muscle in the diabetic mice, suggesting that improvement in IR is essential for enhancing glucose uptake in muscle tissue and improving overall metabolic control [33]. Secondly, insulin can enhance blood flow to the microvasculature in muscle thus improving the access of glucose and insulin to the myocytes to augment glucose disposal [34]. Insulin plays a critical role in causing the redistribution of blood flow from nonnutritive to nutritive capillary networks to improve nutrient delivery to skeletal muscle in response to nutrient consumption. In the muscle and the fat, the blood vessels are the rate‐limiting step for the uptake of insulin [35]. Aging is associated with progressive declines in macro‐/micro‐vascular function; the vasculature becomes less responsive to insulin resulting in lower insulin‐stimulated microvascular blood flow, Microvascular insulin resistance is antecedent and a contributor to impairments in muscle glucose uptake [36]. Thirdly, chronic inflammation contributes to insulin resistance, and skeletal muscle as a secretory organ involves myocyte secretion of inflammatory molecules. Local muscle inflammation may alter myocyte insulin sensitivity via paracrine or autocrine effects [37]. The muscle tissue of patients with IR is in a constant state of inflammation, elevating the risk of muscle atrophy [38], and the reduction or inhibition of inflammation is mostly associated with improvements in insulin resistance and metabolic functions in animal models [39].

The present study provides compelling evidence for a significant association between insulin resistance, particularly obesity‐related metabolic dysregulation and HGS asymmetry. Our findings demonstrated that increased IR, as quantified by TyG‐derived indices, is independently associated with greater prevalence and severity of HGS asymmetry in a dose‐dependent manner. Several key observations merit further discussion. First, the robust correlation observed between the TyG‐BMI index and HGS asymmetry underscores the synergistic contribution of IR and body composition to the development of muscular imbalance. The pathophysiological interplay between IR and BMI contributes to muscular dysfunction through multiple overlapping mechanisms. IR impairs muscle protein synthesis while promoting proteolysis through upregulation of ubiquitin‐proteasome and autophagy‐lysosome systems [13]. Concurrently, adipose tissue expansion exacerbates IR and creates a pro‐inflammatory milieu characterized by elevated TNF‐α, IL‐6 and other cytokines that further disrupt muscle homeostasis [40]. This metabolic‐inflammatory cascade not only accelerates overall muscle loss but may do so asymmetrically, as genetic predisposition, habitual movement patterns or previous injuries create limb‐specific vulnerabilities. Both low and high extremes of the metabolic‐adiposity spectrum predispose to muscular asymmetry; declined TyG‐BMI may indicate sarcopenic obesity or malnutrition with relative preservation of fat mass, leading to impaired muscle quality and regenerative capacity, whereas higher TyG‐BMI represents hyperinsulinemic obesity with amplified inflammatory and metabolic stress [41, 42]. In both scenarios, the combination of impaired insulin signalling and abnormal body composition creates a substrate for disproportionate muscle deterioration. Notably, the persistence of these associations after adjustment for confounding variables emphasizes the independent role of metabolism‐adiposity interactions in muscular dysfunction, suggesting that the identification of TyG‐BMI as a significant predictor of HGS‐A could facilitate early recognition of individuals at elevated risk for musculoskeletal decline. Second, TyG‐WHtR demonstrated the strongest and steepest gradient with HGS‐A, with the overall asymmetry rate 11.6% higher in Q4 than Q1 (*p* < 0.001), highlighting the deleterious effects of central adiposity. Visceral fat drives HGS‐A through the following mechanisms: chronic low‐grade inflammation enhancing muscle catabolism and IR. In addition, intramyocellular lipid accumulation induces lipotoxic stress on mitochondria and calcium signalling, and mechanical stress from abdominal fat shifting and impairing neuromuscular synchronization. The linear trend between TyG‐WHtR and severe asymmetry (prevalence of 4.9% to 5.1%, *p* = 0.042) suggests progressive neuromuscular damage, while the increase in mild‐to‐moderate asymmetry (21.4%–35.1%) reflects early functional compensation failure. These results suggest that central obesity may exert particularly deleterious effects on neuromuscular function through mechanisms such as chronic low‐grade inflammation or altered myokine secretion patterns. This finding aligns with emerging evidence that ectopic fat deposition, particularly in muscle tissue, may directly impair muscle quality and function. In addition, the sex‐specific patterns we observed warrant particular attention. The stronger association between TyG‐BMI and HGS asymmetry in males may reflect gender differences in body composition and fat distribution, while the broader spectrum of significant associations in women could suggest sex‐specific pathways linking metabolic dysfunction to muscular impairment, adding to the growing literature on sexual dimorphism in metabolic‐muscle interactions.

These findings have important clinical implications. The demonstration that readily available metabolic indices can identify individuals at risk for HGS asymmetry suggests potential for early screening and intervention. Given that HGS asymmetry has been associated with increased fall risk and functional decline in older adults, our results support the incorporation of metabolic assessment in comprehensive geriatric evaluations. Study limitations should be acknowledged. First, the cross‐sectional design precludes determination of causality, and we cannot exclude the possibility of residual confounding, such as the influence of medications, as well as the results after adjusting for confounding factors in the current study may also be affected by multicollinearity. Second, our study did not include direct measures of body composition or muscle quality. Future longitudinal studies incorporating advanced imaging techniques would help elucidate the temporal relationship and underlying mechanisms. Third, although CHARLS uses a nationally representative sampling strategy, the overrepresentation of developed provinces may limit the generalizability of our findings, as socioeconomic factors influence both metabolic health and musculoskeletal outcomes. Future studies including populations from less developed regions with varied lifestyles are needed to validate and extend our results.

## 5 Conclusions

In conclusion, our study establishes a significant association between insulin resistance, particularly obesity‐related metabolic dysfunction, and HGS asymmetry. These findings expand our understanding of the metabolic determinants of muscular health and suggest that interventions targeting metabolic syndrome components may contribute to preserving muscle symmetry and function. Further research should explore whether improvement in metabolic parameters leads to measurable changes in HGS asymmetry.

## Author Contributions

**Yuan Zhang** and **Yali Jing** conceived and designed the study. **Yuan Zhang** had access to and verified the data, did statistical analyses and wrote and reviewed the manuscript. **Yuan Zhang and Yali Jing** critically revised the manuscript. All authors had final responsibility for the decision to submit for publication.

## Funding

This work was supported by the National Natural Science Foundation of China Grant Awards (82374554) and Fundings for Clinical Trials from the Affiliated Drum Tower Hospital, Medical School of Nanjing University (2024‐LCYJ‐ZXY‐02).

## Conflicts of Interest

The authors declare no conflicts of interest.
